# Pathways to social integration among homeless-experienced adults with serious mental illness: a qualitative perspective

**DOI:** 10.1186/s12913-024-11678-6

**Published:** 2024-10-04

**Authors:** Melissa Chinchilla, Aaron Lulla, Dylan Agans, Stephanie Chassman, Sonya E. Gabrielian, Alexander S. Young

**Affiliations:** 1 Center for the Study of Healthcare Innovation, Implementation & Policy, Veterans Affairs Greater Los Angeles Healthcare System, 11301 Wilshire Blvd, Los Angeles, CA 90073 USA; 2 Department of Psychiatry and Biobehavioral Sciences, David Geffen School of Medicine, University of California Los Angeles, 760 Westwood Plaza, Los Angeles, CA 90095 USA; 3 VA Desert Pacific Mental Illness Research, Education, and Clinical Center, 11301 Wilshire Blvd, Los Angeles, CA 90073 USA; 4 United States Army, 101 Army Pentagon, Washington, DC 20310-0101 USA

**Keywords:** Social integration, Severe mental illness, Homelessness, Community integration, Homeless services, Veterans

## Abstract

**Background:**

Social integration (i.e., reciprocal interactions with peers and community members) is a notable challenge for many homeless-experienced adults with serious mental illness (SMI). In this study, we examine a range of housing services offered to homeless-experienced adults with SMI and identify the impacts of supportive services on participants’ social integration outcomes, with the goal of improving services in transitional and permanent housing settings for homeless-experienced adults with SMI.

**Methods:**

Through semi-structured interviews with homeless-experienced adults with SMI (*n* = 30), we examine the impacts of housing and service settings on participants’ social integration. Participants received services in a variety of housing settings, including transitional housing with congregate/shared living (*n* = 10), transitional housing with individual quarters (*n* = 10), and permanent supportive housing (*n* = 10).

**Results:**

Participants expressed caution in developing social relationships, as these could pose barriers to recovery goals (e.g., substance use recovery). For many, social integration was secondary to mental and physical health and/or housing stability goals. Individual quarters gave individuals a place of respite and a sense of control regarding when and with whom they socialized. Meeting recovery goals was strongly related to connecting to and receiving a range of supportive services; interviews suggest that proximity to services was critical for engagement in these resources.

**Conclusions:**

Programs serving homeless experienced adults with SMI should seek to understand how individuals conceptualize social integration, and how social relationships can either support or hinder participants’ recovery journey.

**Supplementary Information:**

The online version contains supplementary material available at 10.1186/s12913-024-11678-6.

## Introduction

Social integration remains a critical problem for homeless-experienced individuals with severe mental illness (SMI). Social integration refers to the extent to which an individual engages in social interactions with community members [1], including the size of social networks and the degree to which relationships with others reflect positive support and reciprocity [2, 3]. Social integration has important implications for both mental and physical health [4] and is a critical part of recovery [5], the process through which individuals learn to overcome, manage, or live with the negative consequences of physical illness, mental illness, alcohol or drug use problems, or trauma [6]. While most homeless-experienced populations with SMI are capable of living independently and achieving housing stability [7–9], many experience ongoing struggles with social integration even after being housed [10, 11]. Interventions to improve social integration among homeless experienced populations have had limited success [12], and more work is needed to understand how social integration can be improved.

Homeless-experienced populations have a range of characteristics, including varying degrees of homeless chronicity, mental health symptom burden, and functional impairments. It is generally understood that a variety of services are needed to meet the complex housing, health, and social needs of homeless-experienced individuals [13, 14]. The homeless services system provides a continuum of housing models paired with a range of service types, including transitional housing in congregate and non-congregate settings, and permanent supportive housing (PSH), i.e., subsidized housing with supportive services [15]. Individuals may have different reactions to social and environmental factors based upon previous experiences, preferences, or individual differences [16]. Yet, minimal research examines the relationships between individuals’ psychiatric symptoms and behaviors; housing characteristics and supportive services; and social integration outcomes.

The majority of mental health and housing research has focused on developing services to address symptoms and other individual-level characteristics, rather than examining how housing and service environments can enhance the functioning of homeless services users as a whole [16]. Understanding how individual-level, housing, and service use environments interact can help improve the structure of programs – including case management paradigms, onsite services, and housing design – to better serve homeless-experienced individuals with SMI and support long-term recovery, including improvements in social integration. In this study, we examine a range of housing services offered to homeless-experienced adults with SMI and identify the impact of supportive services on participants’ social integration outcomes, with the goal of improving services in transitional and permanent housing settings. Our study asks, how do differing housing and service models support or hinder improvements in social integration for homeless-experienced adults with SMI?

## Methods

### Conceptual framework

Wong and Solomon developed a conceptual model examining factors influencing community integration, including social integration, among persons with psychiatric disabilities living in supportive independent housing. They theorize that behavioral, support, and housing environments can potentially mediate the impact of personal factors (e.g., demographic factors, symptoms, preferences) on social integration [17]. The behavioral environment refers to programs and policies that affect individuals’ behavior, including the degree of independence offered by housing programs, services available, and program requirements and expectations. The support environment is characterized by the quality of interactions between residents and staff, and whether these are supportive. Lastly, the housing environment includes both the physical and social characteristics of an individual’s residential setting and those of the surrounding neighborhood. These characteristics may include the accessibility of community resources and neighborhood safety [17].

### Study setting

This study was conducted at the Veteran Affairs Greater Los Angeles (VA GLA) Healthcare System, which has the nation’s largest homeless program for Veterans. In 2023, VA GLA engaged over 1,600 unsheltered Veterans and provided financial subsidies for housing for 7,669 Veterans through its PSH program, the Department of Housing and Urban Development-Veterans Affairs Supportive Housing vouchers (HUD-VASH) [18]. VA administers a range of transitional and permanent housing programs on the VA campus and in community-based settings. These programs are targeted towards homeless-experienced Veterans who can live independently and may serve individuals with and without SMI. Transitional housing programs include those that provide individual sleeping quarters (i.e., in a “tiny home” or “pallet shelter”), as well as shared accommodations (i.e., congregate settings) with a range of onsite services. As a PSH program, HUD-VASH enables homeless-experienced Veterans to obtain subsidized, independent housing in the community paired with VA supportive services.

### Participants

Between August 2022 and July 2023, we conducted semi-structured interviews with homeless-experienced adults with SMI (*n* = 30) receiving healthcare at VA GLA. Respondents were identified through research registries of eligible participants, which identified Veterans with an SMI diagnosis with active or prior experience of homelessness. SMI was defined as having a documented diagnosis of schizophrenia, schizoaffective disorder, bipolar, or post-traumatic stress disorders. We purposively sampled respondents across a range of housing services, including transitional housing with congregate/shared sleeping quarters, transitional housing with individual sleeping quarters, and PSH (i.e., HUD-VASH). Participants identified through the research registry were contacted by telephone or, if living on the VA campus, in person, and invited to participate in the study.

An interview guide was developed for this study that examined personal level factors and the potential impacts of behavioral, support, and housing environments on homeless-experienced Veterans’ social integration (see Supplemental File 1). Personal level factors included individual needs and recovery goals. Behavioral environment included degree of independence and availability of services. Support environment was defined by quality of interactions with program staff and other residents. Housing environment was defined by housing design (i.e., shared versus individual living accommodations), community resources, and neighborhood safety. Interviews inquired about how services and housing models impacted participants’ social integration and overall well-being. Interviews were conducted in person or over the telephone, depending on participant preference and availability. Informed consent was obtained verbally, and participants were provided a $30 cash voucher for their participation. Interviews were conducted until thematic saturation was researched and were recorded and professionally transcribed.

### Analysis

We conducted qualitative analysis for health services research [19], using both predetermined and inductive codes. One author (MC) developed an initial codebook comprised of predetermined codes (e.g., housing characteristics, goals, mental health, physical health, substance use, social interactions) based on our conceptual framework and interview guide questions. Using ATLAS.ti 23 software, three authors (MC, AL, and DA) coded the same three transcripts and met to compare and discuss coding results and refine the codebook, adding any emergent themes. Coding discrepancies were reconciled by discussion to achieve consensus. The remaining interviews were split between two authors (MC and AL) for primary coding, and a third author (DA) checked and validated all coding for quality and consistency. Two authors analyzed ATLAS.ti code reports (MC and AL), which differentiated responses by housing type, referring to the fully transcribed interviews when appropriate to identify themes providing insight into how behavioral, support, and housing environments impacted respondents’ social integration and overall well-being. This study was reviewed and approved by VA Greater Los Angeles’ Institutional Review Board.

## Results

### Sample

Table 1 describes the study sample, which included respondents enrolled in three different types of housing services: transitional housing with congregate/shared sleeping quarters (*n* = 10), transitional housing with individual sleeping quarters (*n* = 10), and PSH (*n* = 10). Our sample included sixteen respondents that identified as non-Latino Black/African American, eight as non-Latino White, and one as non-Latino Asian. Our sample included five individuals that identified as Latino, of which four were White-Latino and one was Black-Latino. Respondents were predominantly male (*n* = 25), with five respondents identifying as female, and ranged in age from 30 to 69 years.

**Table 1:** Participant characteristics by housing type

Characteristic	Transitional (congregate/shared)	Transitional (individual)	PSH
Gender	2 Female 8 Male	10 Male	3 Female 7 Male
Age (Range) Age (Mean)	30–69 years of age 49.4 years of age	34–65 years of age 54.6 years of age	33–66 years of age 45.3 years of age
Race/Ethnicity	*Non-Latino*: 4 Black/African American 3 White *Latino*: 3 White-Latino	*Non-Latino*: 3 Black/African American 5 White 1 Asian *Latino*: 1 White-Latino 1 Black-Latino	*Non-Latino*: 9 Black/African American 1 White

We identified notable parallels between respondents’ replies regardless of living arrangement. Therefore, we report our qualitative findings for the sample in aggregate and, where appropriate, note differences. We organized findings by domains in our conceptual model which included personal level factors, and behavioral, support, and housing environments.

### Personal level factors: social integration is secondary to other goals

Motivation for increasing social integration was impacted by respondents’ other recovery goals and often came secondary to improvements in physical health, obtaining permanent housing, or engaging in mental health treatment. Respondents described these goals as critical milestones for feeling prepared to establish new or reestablish previous relationships. When asked about relationships with family, one respondent spoke fondly about his children whom he no longer spoke to but hoped to someday reconnect with. He explained that before being able to reestablish a relationship he wanted to work on his well-being, “first I gotta do some personal things like one, stop smoking. And two, stay on my medication” [Individual transitional housing, Black/African American, Male]. Some respondents also noted wanting to achieve housing stability before focusing on their social lives or even seeing housing as something that could facilitate connections with family and friends by providing a space where others could visit. As one respondent in transitional housing noted, “I will feel more comfortable having my own place…I might be able to invite somebody over” [Individual transitional housing, Black Latino Male]. Additionally, respondents acknowledged that engagement with mental health treatment was a critical step in establishing and/or repairing their relationships. When asked about his relationships with family and friends, a respondent stated, “Well, they’re doing better now because I’m getting healthy… I was embracing being sick. Matter of fact, I didn’t even realize I was sick. I was in denial…So the fact that I’m being honest and dealing with my issues, my life is changing” [Congregate/shared transitional housing, Black/African American, Male].

Many respondents living in transitional housing emphasized the importance of using temporary living accommodations to accomplish personal goals. For some respondents this meant avoiding relationships with other participants that might derail their efforts. For example, respondents working on sobriety frequently chose to distance themselves from residents that were actively using substances. As one respondent in transitional housing noted, “[other residents] have a mental illness on top of either an alcohol problem or a drug problem, so I kind of just stay away because I’ve been sober from my choice of drug…” [Individual transitional housing, White Latino, Male]. Another respondent living in transitional housing expressed empathy for residents struggling with substance use or mental health recovery but shared that working towards his own goals necessitated a level of self-protection; “we’re all in our various stages of the healing process or whatever. But at the same time, I’ve got my own issues and triggers. I ain’t got time for nobody’s bullshit either” [Congregate/shared transitional housing, Black/African American, Male]. As such, stage in recovery for respondents and that of other program participants was an important factor impacting socialization, and many respondents chose not to pursue relationships or actively avoided others.

Obtaining financial goals was also important for establishing and maintaining relationships. Respondents noted that the cost of travel, including paying for public transit or flight tickets, could be difficult to afford. The high cost of travel could make it challenging for respondents to see or reconnect with family and friends as frequently as they would like. Several respondents mentioned trying to save funds for travel to see family or friends. For some respondents, achieving financial goals also made connections with family and friends conceptually easier to establish. For example, being able to buy gifts for special occasions, such as birthdays, or assist family and friends in times of financial need was important to respondents and helped them feel like they were able to contribute to relationships and establish reciprocity. One respondent shared that since receiving VA benefits, he had been able to save money and send gifts to his family and friends, resulting in an increased sense of connection; “it made me feel good. It made me feel like part of the family” [Individual transitional housing, White, Male]. In this way, meeting financial goals or increasing one’s income could contribute to improvements in relationship dynamics.

Meeting recovery goals was important, and, for some respondents, interpersonal relationships felt like a distraction or an active barrier to other goals. As one respondent put it, “right now I’m important to me and I’m trying to do the best that I can to get better…I’m too busy trying to take care of myself right now” [Individual transitional housing, Black/African American, Female]. Other respondents explicitly stated that they chose to stay away from people to avoid additional stressors in their lives, “I have enough crap of my own to deal with. I don’t wanna have to deal with somebody else’s crap” [Individual transitional housing, White Latino, Male]. However, some respondents did acknowledge that a desire to isolate from others was temporary and part of their personal recovery process, “I just don’t feel like opening myself [up] …I don’t feel like it will be forever…” [PSH, Black, Male]. As a result, a focus on other personal goals could delay efforts to work on social integration.

### Behavioral environment: proximity to services supports engagement

For all respondents, achieving their recovery goals was closely tied to accessing a range of VA services. Respondents acknowledged the variety of services available through the VA healthcare system, with several noting efforts to qualify for VA disability compensation, access housing resources, connect with primary care, and engage in mental health services. For respondents living on the VA campus, proximity to services was the most notable factor contributing to treatment engagement, including setting up and attending medical appointments. Respondents reported that being on the VA campus enabled them to learn about VA resources, including housing programs, and to work closely with VA staff to navigate program enrollment. As one respondent stated, “the fact that I am on the VA grounds, and I have access to the VA to take care of my mental health…I meet all my appointments and I’m able to receive the assistance I need in finding permanent housing through the VA” [Individual transitional housing, Black/African American, Female]. Respondents noted that onsite staff were helpful in setting up appointments, reminding respondents about their doctors’ visits, and even encouraging them to seek initial care. Being on the VA campus was particularly impactful for respondents that struggled with transportation or had a propensity for forgetting and missing appointments. As one respondent noted, “It’s so much easier having everything right here. Like I said, I have issues with making appointments and stuff like that. When I was on the street, even just right outside the [VA campus] gates, it was a lot more difficult” [Individual transitional housing, White, Male].

Respondents living on the VA campus were largely connected and actively engaged in VA resources. However, a few respondents did note that the range of services, appointments, and deadlines that accompanied program enrollment could be overwhelming. This was particularly the case for respondents who were new to on-campus transitional housing programs. As one respondent stated, “you have to get yourself worked up to meet deadlines, and there’s a lot of like, tension or stress to just performing all types of activities, whether they be personal errands, or business errands, and then winding yourself back down” [Individual transitional housing, Black/African American, Male]. For some respondents, moving into a new and unfamiliar environment could be stressful and required an adjustment period before they felt they could focus on service engagement. One respondent living in transitional housing on the VA campus illustrated this when speaking about her ability to follow-up with healthcare appointments, “because of so much stress I missed my last appointment with both my doctors, my primary care, and my psychiatrist. But I do have an upcoming appointment this month…which I plan to keep” [Individual transitional housing, Black/African American, Female].

For respondents living in the community at large, transportation, including having one’s own vehicle or being near public transit, was important to ensuring access to care. Transportation access facilitated respondents’ ability to attend care appointments; “it’s convenient as far as public transportation, being not havin’ a car it’s the perfect set up strategically for me to get to different places at different times, especially the VA” [PSH, Black, Male]. However, some respondents were required to travel several hours or long distances to obtain services on the VA campus; “I have to take a lot of time out during the day just to go to an appointment because it takes half a day” [PSH, Black, Female]. Lengthy travel requirements could result in putting off care or needing to take several hours out of the day to attend medical appointments. When respondents did not have competing demands, the length of time needed to travel to the VA for medical care was not an issue and could be seen as an activity to vary their day. However, for respondents that had other commitments lengthy trips to the VA could result in other activities being forgone, “I just have to put things off sometimes to go to one appointment” [PSH, Black, Female].

Lastly, respondents noted that some of their housing programs, both on campus and off campus, attempted to support social integration by facilitating recreational activities and activating communal spaces with residental programming, including service delivery. When asked about onsite activities, most respondents expressed appreciation regardless of whether they chose to participate. Respondents enjoyed onsite activities as a way to explore hobbies and speak with other residents in a space that was structured and often where interactions were facilitated by staff. Even when respondents chose not to participate in onsite activities, they enjoyed having the option to become involved should interest or motivation to engage arise. Respondents also saw these activities as shaping the culture of their place of residence; when discussing social activities onsite, one respondent stated, “I like the open community atmosphere” [Individual transitional housing, White, Female].

### Support environment: building relationships takes time

When asked about the ease of meeting new people, many respondents noted that it was easy to meet others, including neighbors and Veterans engaged in VA services; “it’s pretty easy to meet new neighbors” [Congregate/shared transitional housing, White, Male]. Respondents mentioned meeting neighbors in communal spaces or seeing them out in the local neighborhood. When asked where he was likely to meet people one respondent living in transitional housing stated, “In the room, outside the courtyard, cafeteria. Kind of everywhere” [Congregate/shared transitional housing, Asian, Male]. However, while easy to meet others, interactions did not always translate to friendships. One respondent noted that organized events were a great way to meet other transitional housing residents. However, most residents were not interested in socializing, which he attributed to their focus on their recovery journey; “every now and then, there’s like an outside sponsor that has an event. It’s a good time to sort of socialize with people that stay here, but there’s a lot of stress here… [people] need their time, and they need their space to deal with their own personal issues, so there’s not really too much socializing going on” [Individual transitional housing, Black/African American, Male]. The same participant added, “people are just pretty busy and wrapped up in trying to resolve their personal issues.”

While friendships were difficult to establish, respondents living in transitional housing did note positive interactions with other residents. Interactions could range from occasional greetings, to sharing information, or even trading stories about time spent in military service. However, these relationships often did not continue once individuals moved on to other housing accommodations. When asked if they had been able to develop friendships with others in transitional housing, one respondent shared, “there’s only a few of us left. Everyone has kind of gotten their own places. We’ve seen a lot of people move on…” [Individual transitional housing, White, Male]. Others acknowledged that keeping contact with Veterans that had left transitional housing was difficult, “it’s harder to constantly keep contact, like every day I’m not gonna call” [Individual transitional housing, Black Latino, Male]. Additional respondents noted that it could also be challenging to stay in touch as many lost their phones or changed numbers. Consequently, many respondents in transitional housing did not have the opportunity to form strong bonds with other program participants.

For respondents living in permanent housing, developing relationships with neighbors took time and frequently occurred through happenstance or active engagement with community groups. One respondent living in permanent housing met their neighbor when they were moving into the building, and they offered to help with their belongings. Over the years they became friends, and they frequently drove the participant to their medical appointments. Another respondent living in permanent housing for several years shared that she had befriended her neighbors and joined the local church but noted that for many years while living in housing she experienced notable loneliness – “my life was so lonely” [PSH, Black, Female]. She attributed her current social life to being hospitalized and realizing that she needed to remain on her medications; “I wasn’t on my meds, so I didn’t realize how sick I was…I just realized that I need the medication. I didn’t like myself when I was acting out. People didn’t like me either” [PSH, Black, Female]. Once her mental health was stable, she was able to make a proactive effort to meet her neighbors. Across housing types, it was most common for respondents to describe knowing their neighbors and seeing them in common areas, but not having particularly close relationships. Knowing one’s neighbors contributed to a sense of safety and could encourage participation in activities facilitated in communal areas, but, for most respondents, close relationships were described as developing over extended periods of time and through multiple interactions.

### Housing environment: the impact of individual and shared spaces on social integration

For most respondents it was important to have a space where they could be alone. An individual space was seen as a respite where respondents could work towards their goals and retreat when they needed to decompress or distance themselves from others. This was particularly the case for respondents in transitional housing who at times found it challenging to share spaces with other residents who were actively struggling with mental health or addiction and could feel on edge and overwhelmed by the presence of others. One respondent living in transitional housing with individual quarters noted, “even though we don’t have the keys to the doors, but if I’m in there, I can lock the door, and I have a lot of, I’ve got a lot of mental problems, and that is a huge, huge help” [Individual transitional housing, White, Male]. While discussing being in a shared space, another participant stated, “sharing my own space with somebody else affects my PTSD, very much” [Congregate/shared transitional housing, Black/African American, Male]. This participant added that since moving into shared accommodations he had to change his medication, “to get a higher dosage for the anxiety and for the depression.”

Shared spaces could also lead to conflicts with others. One respondent recounted sharing a room with someone who had been diagnosed with schizophrenia and wrongfully accused him of stealing his belongings. When asked if sharing a room had impacted his well-being, a different respondent shared that there was always the risk of conflict and that sharing a space was “stressful” [Congregate/shared transitional housing, White, Male]. The contrast between living in a shared space and having an individual living arrangement was well illustrated by a respondent that compared his experience staying in congregate shelters to having his own apartment, “you put me in a situation like one of those shelters, I start to get irritable, avoiding people, isolation, and I stop eating and delusional stuff, visions of grandeur. That’s what happens when I’m around a whole bunch of people. But in an apartment, I feel at least the thoughts aren’t so loud, and I think that I can manage” [PSH, Black, Male]. Another respondent also stated that having her own room in transitional housing allowed her to choose whether she wanted to socialize with others – “and I can invite people over, or I can tell people, you know, I need you to leave” – illustrating the impact of individual space on her sense of control [Individual transitional housing, Black/African American, Female].

While respondents largely preferred having their own individual space, there was some tolerance for situations where shared rooming was required. This was particularly true if the number of roommates was limited, respondents got along with their roommates, and/or they viewed shared rooming as short-term and temporary. As one respondent explained, “I personally tend to isolate a lot due to my mental illness…But being the fact that I only have one roommate I deal with it okay. I get along with my roommate just fine. We’re both quiet individuals and reasonably neat so it ends up being just a very decent arrangement, better than I thought it would be” [Congregate/shared transitional housing, Black/African American, Male]. Additionally, when living in shared accommodations it was helpful to have onsite staff mitigate conflicts and model prosocial behaviors, such as clear communication among roommates (Table 2).

**Table 2:** Exemplary homeless-experienced veteran (*n* = 30) quotes by theme

Theme	Representative quote
Social integration as secondary to other goals (personal level)	“…right now, I’m not really focused on [relationships] because I have things that I need to do, is the best way that I could put it. So, it’s like I need to focus on myself and not on other people” [Individual transitional housing, White Latino Male].
Proximity to services supports engagement (behavioral environment)	“I have the VA across the street, and I have a social worker helping me try to get my voucher, gettin’ me an apartment or a house. They’ve been helpin’ me a lot since I’ve been here. They did more for me in those 30 days that I’ve been here than [other transitional housing provider] did in the two years that I was there” [Congregate/shared transitional housing, Black/African American, Male]. “It takes me like a half hour to drive to the VA I go to, but that’s not bad. I have a car” [PSH, White, Female].
Building relationships takes time (support environment)	“You know in passing people are just cordial and polite. And basically that’s it. I mean everyone is doing their own thing” [Individual transitional housing, Black/African American, Female].
Impact of individual and shared spaces on social integration (housing environment)	“I have my own pallet shelter to myself, so I have like my own place to myself, so that’s one, that’s a plus for sure” [Individual transitional housing, White Latino Male].

## Discussion

Interviews with homeless-experienced Veterans with SMI highlight the importance of understanding how homeless-experienced populations with SMI think about and prioritize social integration. While social integration is a core component of recovery in SMI, for study participants social integration often came secondary to other recovery goals which were perceived as foundational for establishing new relationships or reconnecting with family and friends. Meeting personal recovery goals was strongly related to connecting and receiving a range of supportive services. Interviews suggest that proximity to services was critical for homeless-experienced Veterans with SMI, not only because of the ease of access but also the potential role of onsite staff in encouraging individuals to engage in treatment and community programs. While positive relationships and social support may be important for long-term well-being [20], respondents frequently expressed caution in developing relationships as these could be seen as a potential barrier to achieving other personal aims. Without specifying causation or temporality, previous research has also shown that psychosocial function, physical functioning [21], financial stability [22], and satisfaction with one’s housing [23], are all strongly correlated with social integration outcomes. Our interviews suggest that several of these factors may precede social integration for homeless-experienced populations and that it is important to understand how and, if, individuals conceptualize social integration as part of their recovery process. For example, some research shows that, even once participants have moved into PSH, they may not have a strong desire to integrate into their local communities [24] and that social integration may only be weakly associated with life satisfaction [25].

A unique feature of VA GLA is the ability to house homeless-experienced Veterans, in both transitional and permanent housing, on the VA campus. Several respondents living in housing on the VA campus noted the benefits of being near health services and, often, moved to the VA campus to use services and work towards permanent housing placement. Proximity to services meant that respondents’ travel time to appointments was minimal. Additionally, respondents noted that being on the VA campus was helpful in facilitating service use through interactions with VA staff who encouraged and, at times, reminded them to use various services, including primary and mental health care. Other research has also documented the way onsite services can increase engagement in care through fewer cancellations and missed appointments resulting from health challenges or a lack of motivation [26]. Consequently, having services onsite may be impactful for homeless-experienced individuals with SMI, particularly those in the early stages of recovering from health and housing challenges, and can help support future social integration goals.

Respondents in transitional housing were often weary of developing friendships with other residents that were struggling with mental health or substance use problems. An avoidance of close relationships was described as a self-protective mechanism. However, many respondents did note greeting neighbors and engaging in occasional short exchanges. Additionally, respondents viewed organized community events and socializing opportunities favorably. Even when individuals chose not to participate, these events were a reminder that connecting with others was an option and helped create a sense of community. Previous research examining social integration among individuals with SMI has also noted that short interactions with neighbors can have important impacts on experiences of isolation and loneliness [27]. This may be due to the overall sparseness of social networks, but these short interactions can also be the beginning of deeper relationships. In fact, several respondents that had lived in their local communities for longer lengths of time noted meeting neighbors through happenstance in common areas of their building and developing close relationships overtime through multiple interactions. However, for respondents in transitional housing, the transient nature of their living arrangements often meant that contact with other residents was lost and that relationships that started off as acquaintances rarely developed further, e.g., into friendships. As such, transitional housing programs may consider ways to continue to facilitate the development of relationships among participants even after they move into permanent housing. This may include inviting former residents that have obtained permanent housing to onsite community events. Not only would this help facilitate ongoing connection between participants, but it may provide peer support for others working towards housing stability. Future research may also examine longitudinal changes in social integration among homeless-experienced populations with SMI, and the impact of weak versus strong social ties on individual well-being. This research may also seek to develop a better understanding of how social integration is conceptualized by homeless experienced populations with SMI, when individuals are ready to work towards social integration, and/or the role that social integration potentially plays in recovery goals, including as a motivating factor.

Lastly, respondents in our study expressed a preference for living arrangements that allowed for privacy but that also provided the opportunity to engage with others in communal spaces. Other studies have found similar preferences among homeless-experienced persons with SMI [26]. In part, having one’s own space allows individuals to distance themselves from situations or interactions that may exacerbate mental health symptoms [28]. In addition, it can give individuals a sense of control [29]. While independent, permanent housing is the preference for homeless-experienced individuals with SMI [30, 31], interviewees requiring temporary housing arrangements expressed a desire for transitional housing with individual living arrangements over congregate settings. Transitional housing with individual quarters (e.g., tiny home or pallet shelters) may be more resource intensive than shared arrangements but can result in positive experiences for participants. These programs are still relatively rare but are of growing interest to policy makers. Furthermore, as noted by respondents, when shared rooming is the only option, how roommates are assigned, and the way staff support these arrangements can be critical to avoiding negative participant experiences. Onsite staff may help emphasize the temporary nature of shared arrangements, assist participants in findings spaces for brief periods of solitude, and model positive social behaviors, such as open communication, which can help participants enhance their social skills and maintain positive relationships with roommates.

While providing important insights into social integration for homeless-experienced populations with SMI, our study had several limitations. Our study did not differentiate between familial versus non-familial ties or establishing new relationships versus reestablishing previous relationships, which may be prioritized differently by individuals [32]. Our study also did not differentiate between SMI diagnoses or include other diagnoses, such as mood, personality, or substance use disorders, which may also be associated with social integration outcomes. Further, many homeless-experienced individuals with SMI are transdiagnostic and may be struggling with more than one mental health concern. Additionally, we do not discuss the impact of trauma on social integration and how these experiences might shape how individuals view relationships. Past experiences will undoubtedly impact whether relationships are viewed as potentially additive or a distraction from personal recovery goals. Lastly, our study focused on a unique population – homeless-experienced Veterans – and included participants living on a VA campus that provides a range of onsite housing services (i.e., transitional and PSH); this “service rich” environment may not be easily replicated by non-VA providers or other VA’s that lack resources to create onsite housing options. More generally, Veterans have access to a range of services and resources that may not be as easily accessible to civilian populations. It is widely recognized that VA has heavily invested in addressing Veteran homelessness, resulting in an influx of housing and health services and an emphasis on housing first policy (i.e., permanent housing without any preconditions) which has decreased Veteran homelessness at significant rates when compared to the civilian population [33]. While all of our study findings may not be transferable, our study offers important learnings about social integration outcomes among homeless-experienced populations with SMI including how personal goals may impact social integration in different housing and service environments.

## Conclusions

Overall, the results of our study are aligned with previous work on social integration among individuals with SMI and highlight the importance of homeless programs that start with an understanding of an individual’s recovery goals. Programs serving homeless-experienced adults with SMI should also seek to understand how individuals conceptualize social integration, and how social relationships can either support or hinder participants’ recovery journey [34]. Additionally, programs with onsite services should be made available for individuals who desire proximity to health services and may otherwise face challenges to service engagement. Lastly, our study highlights the importance of individual space for homeless-experienced individuals with SMI. Individual space can give individuals a place of respite and a sense of control regarding when and with whom they socialize.

## Supplementary Information

Supplementary Material 1.

## Data Availability

The datasets generated and analyzed during the current study are not publicly available due to reasons of sensitivity but may be available from the corresponding author, with approval from the Department of Veteran Affairs, on reasonable request.
