# Supplementary material for: Pathways to social integration among homeless-experienced adults with serious mental illness: a qualitative perspective
Source: BMC Health Serv Res. 2024 Oct 4;24:1180. doi: 10.1186/s12913-024-11678-6 (PMC11451229; doi:10.1186/s12913-024-11678-6)
Supplement: Supplementary file 1 — Supplementary Material 1. [file 12913_2024_11678_MOESM1_ESM.docx]

**Supplemental File 1:** Social Integration Among Formerly Homeless Veterans with Serious Mental Illness (SMI)

**INTERVIEW GUIDE**

**Housing**

I’d like to start by asking you a couple of questions about where you live.

1. Can you tell me a bit about where you are currently living? What is it like?
   1. Is your living arrangement shared or do you live by yourself?
   2. How long have you lived there? *(If they have lived in place of residence for less than a month, assess if they feel more comfortable answering following questions about previous place of residence*)
   3. What do you like/dislike about where you currently live?
   4. Probes:
      - 1. How about the amount of space you have?
        2. How about your proximity to health services?
        3. How about your surrounding community?
        4. Do you feel safe? Why or why not?

I’m going to be asking you a variety of questions. I would like to focus them on the situation on where you are currently living.

1. I want to understand how where you are currently living affects your quality of life. To start, can you tell me how where you are currently living has impacted your financial stability?
   1. How has it affected your physical health?
   2. How has it affected your mental health?
   3. Probes:
      1. Has availability or lack of availability of VA services had an impact?
      2. Has availability or lack of availability of community resources had an impact? (e.g., transit, stores, recreational spaces)
      3. Has having your own space or sharing a space had an impact?
      4. Have your social relationships with people that live near you had an impact?

**Social Relationships**

Next, I’d like to ask you some questions about your social relationships.

1. Tell me about the people that live near you? What are they like?
   1. Would you say they are different or similar to you? How so?
      1. Probe:
         1. Are they Veterans? Tell me what it’s like to live close to Veterans (or non-Veterans)?
         2. Have they also experienced challenges with housing? Tell me what it’s like to live close to people that have experienced housing changes similar to you (or that have not experienced housing challenges)?
   2. Is it easy or difficult to meet your neighbors? How so?
   3. Probes:
      1. Can you tell me about the places where you are likely to interact with your neighbors?
2. Tell me how often do you leave your community (or the VA campus) to visit friends or family in other neighborhoods?
   1. Can you tell me how you go about visiting?
   2. Probes:
      1. How do you get there? (e.g., bus, car, walk)
      2. How far is it?
3. Tell me about the people that are important in your life?
   1. What kind of support do these people provide to you? (e.g., financial, emotional (e.g., someone you could go to for advice if you had a personal problem), spiritual)

(*Note: If participant does not differentiate, inquire if people mentioned are family, friends, or program staff*)

- 1. IF THEY MENTION FRIENDS ASK, how did you meet these friends people?

1. How important is it for you to develop new relationships? Can you tell me why?
   1. How important is it for you to strengthen your current relationship? (for example, spending more time with family or friends, or reconnecting with people you’ve lost touch with?)
2. Lastly, thinking about your social relationships, how do you prefer to interact with people and why? (e.g., in person, by telephone, text, social media)
3. How often (*Daily, Weekly, Less than once per week*) do you communicate with family or friends,
4. In person?
5. Through telephone calls?
6. Texting?
7. Through social media?

**Conclusion**

I have covered all the questions that I had, and I really appreciate your time and insights. Is there anything else you would like to share?

Thank you so much for participating in this interview. For your participation you will receive a payment voucher of $30.
